# Natural Chemical Composition of Commercial Fish Species: Characterisation of Pangasius, Wild and Farmed Turbot and Barramundi

**DOI:** 10.3390/foods5030058

**Published:** 2016-08-30

**Authors:** Monika Manthey-Karl, Ines Lehmann, Ute Ostermeyer, Ute Schröder

**Affiliations:** Department of Safety and Quality of Milk and Fish Products, Max Rubner-Institut, Federal Research Institute of Nutrition and Food, Palmaille 9, 22767 Hamburg, Germany; Ines.Lehmann@mri.bund.de (I.L.); Ute.Ostermeyer@mri.bund.de (U.O.); Ute.Schroeder@mri.bund.de (U.S.)

**Keywords:** aquaculture, proximate composition, minerals, fatty acids, free amino acids, citric acid, phosphates, water content

## Abstract

To comply with the relevant legal requirements and correct labelling, it is necessary for business operators and inspection authorities to know the natural characteristics of the raw material. This study gives a comprehensive overview of muscle flesh composition of farmed and wild Atlantic turbot (*Scophthalmus maximus*) and barramundi (*Lates calcarifer*) and of farmed pangasius (*Pangasianodon hypophthalmus*). The proximate composition, di- and triphosphates and citric acid values are presented in order to evaluate possible indicators for a hidden treatment during processing to fillets. All moisture contents were ≤80%. Even for pangasius, protein values for deep skinned fillets of ≥18% were determined. Only small quantities of naturally occurring citric acid (up to 0.03 g·kg^−1^) were detectable. The lipid content was the most varying main component within the different species, ranging between 1.2% to 2.0% and 0.3% to 3.0% for farmed turbot and barramundi, respectively. Pangasius flesh had a mean lipid content of 7.8%. Trimming and separation of the red layer reduced the lipid content of the commercially sold white-flesh fillets to 2.7% to 3.5%. Fatty acids profiles, free amino acids, and minerals were analysed to show the nutritional quality of the aquaculture fish species and compared to wild turbot and barramundi. Despite some natural variation, these components can be considered as comparable.

## 1. Introduction

More and more fish are being raised on farms; globally it accounts almost for 50% of the fish consumed [1]. Like other goods, aquaculture products must pass through distribution channels in order to reach the consumer as the end-user. In Europe, improved logistics and effective intermediaries have shortened the chain so that imported fish are brought directly from the source by the wholesaler. Higher prized fish are often marketed fresh and whole, like Atlantic turbot (*Scophthalmus maximus*) from aquaculture plants in Chile or Spain. As its wild counterparts, this flatfish species is a relatively scarce “gourmet” fish. European aquaculture production of Atlantic turbot has grown steadily up to more than 12,000 t in 2012 [2] and extended worldwide to 77,000 t in 2013 [3]. Barramundi or giant perch, (*Lates calcarifer*) is indigenous to the waters of South East Asia and Northern Australia and tolerates a wide range of salinities which allows for cultivation in both, fresh and seawater [4,5]. It is a rather new species on the European market, whereas pangasius (*Pangasianodon hypophthalmus*), mainly imported from Vietnam, has been popular for several years. However, it has also been the subject of criticism because of very different qualities [6,7,8]. Both species are mostly provided frozen as fillets. Barramundi and Atlantic turbot rank among the species with high international reputations as fish with premium eating qualities, whereas pangasius belongs to the low cost products on the German market.

As a valuable source of protein, essential fatty acids, and micronutrients, fish is important for diversified and healthy diets. However, several publications and annual reports of federal investigation offices for food in Germany often note appreciable changes at the retail level, mainly resulting from too high water content [6,9].

In order to verify the conformance with legislation and to comply with correct labelling requirements, it is important for business operators and inspection authorities to know the natural composition of the product. Consumers’ growing reservations about chemical ingredients, such as phosphates, have led to an increasing number of products with “chem-free” labels. Therefore, the aim of this study was to analyse and to determine what differences (if any) exist between untreated raw muscle flesh of pangasius and wild and farmed turbot and barramundi for proximate composition, fatty acids, minerals, amino acids, citric acid, and phosphate concentrations.

## 2. Materials and Methods

### 2.1. Sample Procurement

All fish were delivered adequately cooled to the Max Rubner-Institut in Hamburg, Germany, where the chemical analyses were carried out. Raising conditions and transport were not assessed nor controlled in this study.

Turbot: Organised by a German wholesaler, whole fresh turbots were directly transferred from four different aquaculture plants within five days to the institute in polystyrene boxes in ice by air from Chile or by truck from Spain, respectively. Wild turbot was caught in the North-East Atlantic and delivered gutted via Lemvig, Denmark. Upon arrival, all specimens were individually weighed, filleted, and deep frozen.

Barramundi: Gutted plate size saltwater barramundi were bought directly from an Australian aquaculture company; fresh fillets of wild barramundi were purchased from an Australian seafood retail outlet. All samples were delivered frozen by air cargo. Frozen skinned fillets from farmed and wild barramundi were imported from Vietnam and obtained from a wholesaler.

Pangasius: Eviscerated headed fish, skin-on cutlets, and European Union (EU) organic certificated skinned fillets were imported frozen from Vietnam and obtained from a wholesaler.

All fish were stored in the same storage chamber, except those for the investigation of citric acid and condensed phosphates which were kept at −80 °C. For chemical analysis, samples were thawed in a refrigerator overnight at 4 °C. Barramundi fillets without skin were prepared from the Australian fish. In the case of whole pangasius, one fillet of each specimen was skinned without removing the red muscle layer, the other was deep skinned. Both were trimmed. From pangasius cutlets, only the white flesh was used.

The resulting samples (including drip loss, if any) were homogenized. Chemical tests of the homogenates were done in duplicate.

### 2.2. Chemical Analyses

#### 2.2.1. Proximate Composition, Total Volatile Basic Nitrogen (TVB-N) and pH

Percent moisture and ash content were determined by drying samples of approximately 5 g at 105 °C for 12 h to a constant weight, followed by ashing at 550 °C [10].

Percent nitrogen was measured by modified Dumas method, using a LECO TruSpecN (LECO Instruments GmbH, Mönchengladbach, Germany). Percent protein was calculated by multiplying percent nitrogen by 6.25 [11]. Total lipids were extracted, using the method described by Smedes [12] with modification by Karl et al. [13] which included extraction of lipids from the homogenised sample with a mixture of isopropanol and cyclohexane. Percent salt (NaCl) was obtained by potentiometric titration of an aqueous sample solution with 0.1 N AgNO_3_ solution, applying the method of Karl et al. [14]. The total phosphorus content was estimated photometrically in the nitric acid extract of the ash, according to a modified official § 64 LFGB German method to determine phosphorus in meat [15]. The colorimetric reaction was measured at 430 nm. The pH value was determined in minced samples (homogenised with one part of deionised water). A perchloric acid extract of 20 g homogenised muscle flesh and 180 mL 6% (*w*/*w*) perchloric acid was used after filtration for the determination of TVB-N [16]. Certified reference materials were used as quality control for the accuracy of pH, protein, phosphorus, and chloride measurements.

#### 2.2.2. Mono-, Di- and Triphosphates

Monophosphates and the condensed phosphates di- and triphosphates were analysed by ion chromatography (IC) and conductivity detection by means of a suppressor technique, according to the method of Kaufmann and his working group [17,18]. The IC conditions were as follows: gradient elution with 50 mM sodium carbonate and 50 mM sodium hydrogen carbonate buffer, using the stationary phase Metrosep A Supp 5–100 (Metrohm, Filderstadt, Germany), combined with two pre-columns, Metrosep A 4/5 Guard (Metrohm) and Hypercarb (4.6 × 10 mm, 5 µm; Fisher Scientific, Schwerte, Germany). The sample preparation included heating of 5 g homogenised muscle tissue in 250 mL deionised water at 100 °C for a few minutes to stop phosphatase activity. Clarification of the extracts was achieved by centrifugation and filtration (syringe filters 0.2 or 0.45 µm), followed by injection of 10 µL into the HPLC-system. The limit of detection (LOD) for di- and triphosphates is 0.01 g·kg^−1^. Recovery rates of 95.7% to 101.6% for diphosphates and 97.5% to 99.2% for triphosphates were determined by standard addition in the range of 0.5 to 2.0 g·kg^−1^ and verified the accuracy of this method.

#### 2.2.3. Citric Acid

For the citric acid determination, our own laboratory procedure was developed [9]. The isocratic HPLC approach for citric acid was carried out on the column Synergi Hydro RP 80A, 4 µm (250 × 4.6 mm) with the pre-column AQ C18 (4 mm × 3.0 mm) (both Phenomenex, Aschaffenburg, Germany), and UV detection at 220 nm. The mobile phase, consisting of 20 mM potassium dihydrogen phosphate, pH = 2.5, was adjusted to a flow rate of 0.4 mL·min^−1^. After preparing aqueous extracts (100 mL) from homogenised samples (5 g), Carrez I and II (2 mL each) were added, and precipitated proteins were removed with a pleated filter. Before injecting into the HPLC (20 µL), extracts were further purified by syringe filters (0.2 µm). An LOD of 0.005 g·kg^−1^ as well as a limit of quantification (LOQ) of 0.02 g·kg^−1^ were established for citric acid. Recovery rates of 89.0% and 89.3% by standard addition of 0.5 g·kg^−1^ and 1.0 g·kg^−1^ citric acid in fish muscle tissue confirmed the accuracy of this method.

#### 2.2.4. Free Amino Acids, Including Taurine

Free amino acids were determined in the perchloric acid extracts of the individual fish samples, according to a modified method of Antoine and co-workers [19].

In brief, for sample deproteinisation, 10 g of minced fish fillet were homogenised with 90 mL 6% perchloric acid (*w*/*w*) and subsequently filtrated. HPLC determination of the free amino acids was performed in the diluted extracts (1:10 up to 1:500). After pre-column derivatisation with o-phthaldialdehyde (OPA) the eighteen amino acids were separated on a reversed-phase column by a solvent gradient and then quantified by fluorescence detection, using the internal standard method with 2-aminobutyric acid [20].

The LOQ was 1 mg·(100 g)^−1^ fish tissue (wet weight) for each amino acid. A commercial amino acid standard for fluorescence detection (Sigma-Aldrich A 2161, Sigma-Aldrich, Merck, Darmstadt, Germany) was used as quality control.

#### 2.2.5. Fatty Acid Profiles

Fatty acid methyl esters (FAME) were obtained from 100 mg of extracted lipids by trans-esterification with methanolic potassium hydroxide [21]. Fatty acid composition was determined, according to the German Society for Fat Science’s (DGF) standard method [22].

FAME analyses were performed on a Hewlett Packard 6890 gas chromatograph (Agilent Technologies, Santa Clara, CA, USA) equipped with a split injection port, auto sampler, FID, and a 60-m fused silica capillary column DB-23 (60 m, 0.32 mm × 0.25 µm, Agilent). Injector and detector temperatures were 250 °C and 300 °C, respectively. The oven program was as follows: 140 °C for 5 min, to 160 °C at 2 °C·min^−1^, 10 min isothermal, 1 °C·min^−1^ to 180 °C, 5 min isothermal, 20 °C·min^−1^ to 250 °C and finally 10 min isothermal. Evaluation of chromatograms was performed with an Agilent Chem-Station. Fatty acids were identified by comparison of retention times to authentic standards. The fatty acid contents in the range of 14:0 to 22:6 n-3 were shown as peak areas of all measured fatty acids (Details for calculation: [23]).

#### 2.2.6. Mineral Element Analysis

Analytical details are described by [9]. In brief, 2 g muscle homogenate were digested in a mixture of 4 mL 65% nitric acid (*w*/*w*) and 1 mL 30% hydrogen peroxide (*w*/*w*) in a closed tetrafluormethaxil quartz vessel of a temperature time programmed Milestone ultraCLAVE III digestion system (Milestone SRL, Sorisole, Italy). Sodium (Na), potassium (K), calcium (Ca), magnesium (Mg), and Zinc (Zn) were determined by a contrAA^®^ 700 high-resolution continuum source atomic absorption spectrometer with air-acetylene flame and equipped with an auto sampler (Analytik Jena, Jena, Germany). Instrumental parameters were based on the cookbook methods for the contrAA^®^ 700 CS. Adequate calibration solutions were prepared using standard solutions (Titrisol, Merck, Darmstadt, Germany). Selenium (Se) and arsenic (As) were analysed by the continuous flow hydride system of the contrAA^®^ 700.

All samples were determined in duplicate. Blanks were digested within each run.

The commercial reference material IAEA-407 (International Atomic Energy Agency, Vienna, Austria) was used to validate the analytical methods and as quality control. Several participations in NAEL inter-laboratory comparisons on determination of the analysed elements in fish material confirmed good results. The mean values obtained for analytical recovery and the LODs were 81% and 0.09 mg·kg^−1^ (Na), 93% and 0.07 mg·kg^−1^ (K), 89% and 0.1 mg·kg^−1^ (Ca), 87% and 0.01 mg·kg^−1^ (Mg), 88% and 0.01 mg·kg^−1^ (Se), 87% and 0.02 mg·kg^−1^ (As), and 89% and 0.07 mg·kg^−1^ (Zn), respectively.

### 2.3. Statistical Evaluation

One-way ANOVA was applied for statistical analysis (SigmaStat version 3.5, Systat software Inc., San Jose, CA, USA).

## 3. Results and Discussion

### 3.1. Proximate Composition, pH and TVB-N

The proximate composition of all analysed turbot, barramundi and pangasius samples is shown in Table 1, Table 2 and Table 3. Muscle pH (6.4–6.9) and TVB-N values (9.9–20.4 mg·(100 g)^−1^) were typical for good quality fish. The ash content of all specimens was between 0.8% and 1.3%. The mean water content in the farmed fish was ≤80%. NaCl content of untreated muscle is low [24]. Fish fillets washed with salt water can have slightly higher concentrations. Calculated from the analysed Na values, a corresponding NaCl content of <0.1%–0.4% can be expected and was confirmed in this study with the exception of turbot from a Chilean aquaculture farm (0.5% ± 0.1%).

Turbot: Wild individuals contain lipid contents near 1% which is in agreement with the results shown here in Table 1 for turbot from the North East Atlantic (1.0%). Only slightly higher lipid contents were found in cultured turbot (1.2%–2.6%), which is consistent with the results obtained by Sérot et al. [25]. Between the farmed individuals, the variation of water (78.7–79.2, *p* < 0.05) and protein (18.9%–20.3%) was small.

Barramundi: Fillets (Table 2) showed clear differences in lipid content among different origins (*p* < 0.05) with higher mean amounts in barramundi from Australia which included the belly flaps, containing higher levels compared to the tail and dorsal parts [26]. These findings are in agreement with Karl et al. [27] who also analysed low lipid content between 1.3% and 1.7% in imported frozen farmed fillets. Protein and moisture values were found to be 18.2% to 20.8% and 75.7% to 80.2%, respectively, inclusive of wild specimens. The significantly higher level of 80.2% water in combination with a citric acid content of 0.188 ± 0.060 g·kg^−1^ could not be traced back to explainable causes.

Pangasius: Frozen fillets are traded in very different qualities, indicated by low protein and high water content in the flesh, often combined with elevated pH values up to 8.5 (our own unpublished results) [6,28,29]. All analysed samples (Table 3) had mean moisture values <80% and a pH < 7. The protein share ranged between 17.2% and 18.7%. Water addition up to 82%–83% can reduce the protein content to less than 14%, as estimated by Karl et al. [6]. The results also show that content and variability of moisture and lipid were distinctly (*p* < 0.05) influenced by the different skinning techniques. Pangasius flesh (edible part without belly flaps) had a mean lipid content of 7.8%. Trimming and separation of the red layer by deep skinning are common practices in Vietnamese companies and reduce the lipid content of the commercially sold white-flesh fillets significantly. Values of 2.7% to 3.5% lipid for deep skinned fillets are comparable with reported data between 1% and 3% [6,28,30].

### 3.2. Macro and Micro Minerals

The dominating macro mineral element in fish tissue is K followed by Na. Both can vary in a large range between 2500 and 5000 mg K·kg^−1^ and 300–1500 mg Na·kg^−1^, respectively [31]. Compared to the low Na muscle contents in barramundi from Australia handled as whole fish (336–633 mg Na·kg^−1^), higher amounts in barramundi fillets from Vietnam were found (1255–1770 mg Na·kg^−1^), confirming similar results of Karl et al. [27]. Potassium (2590–3858 mg·kg^−1^) remained in its natural range. The level of Mg in the raw and boneless muscle is higher than that of Ca. Summarising the results of this study, Mg ranged from 194 to 295 mg·kg^−1^ which is comparable to reported data of 150–300 mg·kg^−1^ for white finfish [32]. Naturally low Ca values can potentially be increased by incomplete deboning [33]. Twenty to 281 mg Ca·kg^−1^ were estimated. Apart biological factors a high variability caused by bone fragments is also due to phosphorus (P). The natural occurring P in the flesh (estimated as P_2_O_5_) is on average 2.2 g kg^−1^ (≙ 5.7 g P_2_O_5_·kg^−1^), covering a range between 1.0 and 4.0 g P·kg^−1^ (≙ 2.6–10.4 g P_2_O_5_·kg^−1^) [34]. For the farmed species values between 3.1 and 5.3 g P_2_O_5_·kg^−1^ were found.

As an essential micronutrient, Selenium plays a vital role in human health and is incorporated into proteins to yield selenoproteins, which are important antioxidant enzymes. Fish is an important and highly bio available source of dietary selenium [35]. Zinc, too, is essential for humans. It is the most abundant trace element. The results for Se and Zn cover the expected range for these elements in fish [36], except for the lower Se values for farmed Australian barramundi and pangasius.

Seafood can have naturally high levels of arsenic (As) and is known as the main contributor in the diet [37]. The EFSA [37] reported 0.1 to 1.8 mg·kg^−1^ as the range for the average total arsenic concentrations in a mix of marine and freshwater fish and other seafood. Most levels found in farmed fish were in the lower or middle range. Affected by the vegetarian dominated diet, pangasius had the lowest amounts (<0.02 mg·kg^−1^) in the muscle flesh. The sample of wild turbot contained 4.6 mg As·kg^−1^ and fits with the high amounts reported for body dwelling fish species [38].

### 3.3. Citric Acid

According to European legislation, food additives such as citric acid and its sodium salts are allowed without quantitative limitations (*quantum satis*) in frozen fish fillets with the requirement of correct labelling. There is little information in the literature about naturally occurring citric acid in aquatic animals. Chew and Ip [39] detected in the lateral muscle of mudskippers (*Boleophthalmus boddaerti*) approximately 44 mg citric acid kg^−1^ and Piironen and Hyvärinen [40] found average concentrations from 5.49 to 45.13 mg citric acid/100 mL in the seminal milt plasma of various fresh water fish like *Perca fluviatilis*, *Salmo gairdneri*, and *Coregonus lavaretus*. In the case of controlling correct labelling, competent authorities need reliable information about naturally occurring concentrations in fish muscle. This knowledge enables authorities to assess citric acid content in a fish fillet as a natural background, a cross-contamination or as an intentional addition to affect fish quality. In the last few years, different research groups have carried out investigations to improve the shelf life of different fish species and products with the addition of organic acids such as citric acid and/or its sodium salts used in dipping solutions [41,42] or in icing mediums [43,44]. Depending on the mentioned approaches, preliminary results of the analysis of various commercial products showed that the amount of citric acid might be low (not more than 0.1 g·kg^−1^) or higher up to 1 g or 3 g·kg^−1^ .In the present study the results ranged from below the levels detectable by the analytical method (LOD = 0.005 g·kg^−1^) and 0.03 g·kg^−1^. However, the barramundi fillets from Vietnamese aquaculture with a concentration of 0.188 ± 0.060 g·kg^−1^ on average seem to be an exception. Although declared as untreated, it could not be excluded that these concentrations were caused by a manipulation during filleting. The other low values for turbot, barramundi, and pangasius comply with the results of the Swiss State Laboratory of the Canton Bern [45,46]. Here the Swiss scientists found concentrations of citric acid lower than 0.1 g·kg^−1^ in different processed frozen fish fillets and crustaceans. They concluded that citric acid concentrations must be of natural origin, although they could not finally clarify, whether traces of citric acid were natural or from carry-over effects during production. The presented data, relating to unprocessed fish muscle tissue, confirm their assumption that only small quantities of citric acid are detectable in some fish species such as in turbot with concentrations of >0.005 g·kg^−1^ to 0.03 g·kg^−1^ (Table 1) and as in barramundi with concentrations of <0.02 g·kg^−1^ (Table 2).

### 3.4. Phosphates

Condensed phosphates could not be detected or assessed by using the photometric method for total phosphorus (Table 1, Table 2 and Table 3). Instead, IC analysis is the method of choice to control the potential addition of polyphosphates such as di- or triphosphates. Higher water content in fish fillets can often be attributed to the application of condensed phosphates due to their water binding effects. However, none of the analysed fish samples in this study contained these additives, although the barramundi fillets from Vietnam show comparably higher moisture values than barramundi from Australia.

### 3.5. Fatty Acid Profile

In turbot samples (Table 4) the fatty acid (FA) profiles within the farmed specimens were similar, but differed mainly from the wild counterparts. Apart from comparable amount of about three quarters of unsaturated FA in all samples, the percentage content of the important omega-3 polyunsaturated fatty acids (PUFA) docosahexaenoic fatty acid (C22:6n-3, DHA) and eicosapentaenoic fatty acid (C20:5n-3, EPA) was significantly higher in the fillets from wild fish (22.6%, *p* < 0.05). In the muscle of the wild samples, significantly lower contents for oleic acid (C18:1n-9) and linoleic acid (C18:2n-6c) and higher amounts for gondoic acid (C20:1n-9) were found (*p* < 0.05). As a result of the different feeding strategies and independent of origin, the main differences in the farmed groups were found in the content of linoleic acid (C18:2n-6c).

The fatty acid profiles of barramundi (Table 5) were characterised by high contents of saturated FA (36.9%–43.5%), except farmed fish from Australia (28.0%) that had monounsaturated FA (43.6%) nearly twice as high compared to the other specimens (24.6%–25.6%). A detailed look at the wild fish showed higher contents of pentadecanoic acid (C15:0) of 4.2% and palmitoleic acid (C16:1n-7) of 9.6% in Australian samples compared with 0.7% and 4.6%, respectively, in those from Vietnam. On the other hand, the Australian wild specimens had lower amounts of DHA with 4.1% vs. 13.3% in those from Vietnam. Looking at the FA composition of the Australian aquaculture samples, they are characterised by high amounts of oleic acid (18:1n-9) and linoleic acid (C 18:2n-6). Compositions found for Vietnam aquaculture samples correspond well to findings of Karl et al. [27].

In all samples, the fatty acid composition of pangasius muscle (Table 6) was characterised by low amounts of PUFA, less than 20%, and high shares of saturated and monounsaturated FA. Palmitic acid (C16:0) and oleic acid represented nearly two thirds of all analysed FA. Deep skinning did not influence the nutritional value, whereby EPA and DHA contents were both unusually high compared to the analysed cutlets and organic fillets (*p* < 0.05).

### 3.6. Free Amino Acid Profile

Amino acids are not only components of all proteins, but also precursor compounds relevant to flavour, taste, and colour of cooked foods. The contents of free amino acids (FAA) in food, especially of the indispensable and conditionally indispensable ones, are also interesting because of nutritional aspects.

Due to their osmoregulatory function, fish and other seafood usually contain more FAA than terrestrial animals. However, the total concentration of FAA in fish muscle is generally low, i.e., between 0.5% and 2%, and can, additionally, be decreased during industrial processing and household preparation. Content and kind of FAA vary according to species and physiology of fish. Factors like seasonal variations, temperature, salinity, and diet influence the concentration of FAA too [47,48]. During the storage of fish, FAA are used by microorganisms as major substrates for growth [49].

The mean amounts of the four most frequent FAA and the sums of the indispensable, conditionally indispensable, and all analysed FAA in muscle tissue of turbot, barramundi, and pangasius are compiled in Table 1, Table 2 and Table 3. The mean total contents in the three species ranged between 144 and 519 mg·(100 g)^−1^ fish fillet with pangasius showing the lowest values. All values varied more or less between single specimens within the same species. The four most frequent FAA accounts for at least 65% up to 90% of the whole FAA-pool.

With one exception, taurine was the most prevalent compound in all tested fish samples. The amino sulfonic acid taurine is of importance for various physiological processes in humans. For example, taurine is beneficial for cardiovascular health, cell membrane stabilisation, and immune defence enhancement; it reduces blood cholesterol values and has antioxidant properties [50,51]. Taurine is considered as a conditionally indispensable amino acid. During processing of fish, the content of taurine decreased mainly as a result of leaching [52].

Whereas the FAA-pool of cultured turbot tissue consisted on average of 53% taurine, the share in the fillet of wild turbot specimens accounted for three-quarters (Table 1). Altogether, the wild turbots contained a greater percentage of indispensable and conditionally indispensable FAA than the cultured turbots.

In all tested barramundi fillets, alanine, glutamic acid, glycine and taurine were the most important FAA from a quantitative point of view (Table 2). This is consistent with a previous study [27]. However, the percentage composition of the FAA-pool was significantly different for the farmed fish coming from Vietnam and Australia. Especially the farmed Australian barramundi which contained a noticeably high amount of glycine. The low taurine content of these farmed individuals is probably due to a diet with only small amounts of sulphur containing amino acids [47]. Compared to the farmed animals, the percentage composition of FAA in wild barramundi from Vietnam and Australia are much more similar.

As expected, the deep-skinned fillet of whole pangasius contained not only more water and protein, but also a slightly higher amount of FAA than the skinned fillet of the same individual. However, the percentage composition of FAA in the two fillets corresponded (Table 3). Pangasius originating from organic aquaculture contained the lowest amount of FAA. Because of the small amounts of histidine in all fish samples, notable amounts of histamine in stored products are not expected.

## 4. Conclusions

The individual nutritional quality of farmed turbot, barramundi, and pangasius based on minerals, fatty acids, and free amino acids can be considered as comparable. Differences and variations are explainable by natural causes. However, the lipid content varied due to different feeding strategies. The main components, such as moisture and protein, seem to show only little variation in aquaculture species like barramundi and turbot and are comparable to their wild-caught counterparts. All moisture values were ≤80%. Our results clearly show that untreated pangasius has protein values >18%, even in the case of deep-skinned fillets.

It is not possible for foods to always contain the exact composition labelled due to natural variabilities and/or variations from production and storage conditions. Inspection authorities have to struggle with the fact that the products placed on the market are normally far away from the measurable entrance level and its basic composition. For example, most of the frozen pangasius fillets from conventional aquaculture sold on the European market are treated with water-binding additives without adequate labelling. The study shows that, in general, citric acid concentrations in unprocessed fish fillets are below under 0.1 g·kg^−1^.

Although this study is based on a limited number of samples, which may not reflect the full variation of “typical wild” or “typical cultured” fish products, it can provide basic information for fish species that are relatively new to the European market.

## Figures and Tables

**Table 1:** Meat composition: Mean values ± standard deviation of turbot from four different aquaculture farms and of wild turbot (origin North-East (NE) Atlantic).

Origin	Aquaculture Spain		Aquaculture Chile		NE-Atlantic Wild
Number of Fish	8	10	10	10	9
Weight (g)/Length (cm)	988 ± 21/37 ± 2	737 ± 35/34 ± 1	693 ± 88/33 ± 1	663 ± 87/32 ± 1	2333 ± 210/50 ± 2
pH	6.8 ± 0.1 ^a^	6.7 ± 0.1 ^a^	6.8 ± 0.1 ^a^	6.7 ± 0.1 ^a^	6.7 ± 0.1 ^a^
TVB-N * (mg·(100 g)^−1^)	12.6 ± 1.2	14.2 ± 1.0	13.6 ± 1.1	11.7 ± 1.2	17.1 ± 2.3
Moisture (%)	79.2 ± 1.7 ^a,b^	78.7 ± 0.9 ^a^	78.9 ± 0.6 ^a^	78.7 ± 0.4 ^a^	80.2 ± 1.0 ^b^
Lipid (%)	2.0 ± 0.4 ^a^	1.6 ± 0.5 ^a,b^	1.2 ± 0.5 ^b^	1.5 ± 0.3 ^a,b^	1.0 ± 0.6 ^b^
Protein (%)	18.9 ± 0.6 ^a^	20.2 ± 0.4 ^b^	20.3 ± 0.5 ^b^	19.7 ± 0.6 ^a,b^	19.0 ± 0.7 ^a^
Ash (%)	1.0 ± 0.2	1.2 ± 0.1	1.1 ± 0.0	1.3 ± 0.0	1.1 ± 0.0
NaCl (%)	0.3 ± 0.1	0.3 ± 0.1	0.3 ± 0.2	0.5 ± 0.1	0.3 ± 0.1
P_2_O_5_ (g·kg^−1^)	3.1 ± 0.3 ^a^	3.8 ± 0.1 ^b^	3.3 ± 0.2 ^a,c^	3.6 ± 0.2 ^b^	3.4 ± 0.1 ^c^
Citric acid (g·kg^−1^)	<0.02	<0.02 ^1^	0.03 ± 0.01	<0.02 ^1^	<0.02
Diphosphate (g·kg^−1^)	<0.01 ^2^	<0.01 ^3^	<0.01 ^3^	<0.01	<0.01 ^2^
Triphosphate (g·kg^−1^)	<0.01 ^2^	<0.01 ^3^	<0.01 ^3^	<0.01	<0.01 ^2^
Na (mg·kg^−1^)	1162 ± 114 ^a,b,c^	1062 ± 98 ^c^	1106 ±80 ^a,c^	1769 ± 244 ^b^	1084 ± 96 ^a,c^
K (mg·kg^−1^)	2590 ± 283 ^a^	3131 ± 92 ^b,c^	2905 ± 140 ^b,c^	3141 ± 278 ^c^	2836 ± 183 ^a,b^
Ca (mg·kg^−1^)	108 ± 54	23 ± 7	129 ± 53	20 ± 0	90 ± 17
Mg (mg·kg^−1^)	206 ± 15	215 ± 10	194 ± 11	238 ± 10	240 ± 20
Zn (mg·kg^−1^)	7.6 ± 0.6	6.1 ± 0.5	5.9 ± 0.6	5.2 ± 0.4	6.1 ± 1.2
Se (mg·kg^−1^)	0.22 ± 0.02	0.31 ± 0.02	0.26 ± 0.01	0.36 ± 0.02	0.40 ± 0.10
As (mg·kg^−1^)	0.51 ± 0.10	0.82 ± 0.19	0.30 ± 0.06	0.48 ± 0.03	4.60 ± 1.10
*Most frequent free amino acids (FAA) and sums of FAA groups*					
Alanine (mg·(100 g)^−1^)	30.1 ± 3.3 ^a^	35.6 ± 4.7 ^c^	35.2 ± 4.3 ^c^	31.2 ± 3.0 ^a,c^	13.4 ± 1.6 ^b^
Glutamic acid (mg·(100 g)^−1^)	19.4 ± 2.9 ^a^	32.0 ± 5.7 ^b^	23.9 ± 5.4 ^a^	19.5 ± 3.6 ^a^	9.0 ± 2.9 ^c^
Serine (mg·(100 g)^−1^)	18.8 ± 2.8 ^a^	32.3 ± 6.4 ^c^	24.0 ± 7.8 ^a,c^	22.7 ± 4.5 ^a,c^	2.9 ± 1.6 ^b^
Taurine (mg·(100 g)^−1^)	118.6 ± 18.4 ^a^	151.1 ± 24.5 ^c^	217.1 ± 29.1 ^b^	203.7 ± 22.0 ^b^	187.8 ± 15.1 ^b^
∑ indispens. FAA ^4^ mg·(100 g)^−1^)	30 ± 4 ^a,b^	37 ± 7 ^a^	32 ± 5 ^a^	31 ± 8 ^a^	21 ± 9 ^b^
∑ cond. indispens. FAA ^5^ (mg·(100 g)^−1^)	127 ± 19 ^a,c^	158 ± 25 ^c^	227 ± 30 ^b^	215 ± 23 ^b,d^	193 ± 16 ^d^
Total FAA ^6^ (mg·(100 g)^−1^)	236 ± 29 ^a^	314 ± 37 ^d^	376 ± 38 ^b^	353 ± 34 ^b,d^	244 ± 24 ^a,c^

^1^ *n* = 9; ^2^ *n* = 4; ^3^ *n* = 5 fish samples, resp.; ^4^ Indispensable FAA: His, Ile, Leu, Lys, Met, Phe, Trp, Thr, Val; ^5^ Conditionally indispens. FAA: Arg, Tau, Tyr; ^6^ Total FAA: Ala, Asn, Asp, Glu, Gly, Ser, Orn His, Ile, Leu, Lys, Met, Phe, Trp, Thr, Val, Arg, Tau, Tyr; * Total volatile basic nitrogen. Values with different superscripts within a line are significantly different (*p* < 0.05).

**Table 2:** Meat composition: Mean values ± standard deviation of farmed and wild barramundi of different origins.

Species and Origin	Barramundi Vietnam		Barramundi Australia	
	Aquaculture	Wild	Aquaculture	Wild
Number of Samples	10	4	10	10
pH	6.9 ± 0.1 ^a^	6.6 ± 0.1 ^b^	6.7 ± 0.1 ^b^	6.4 ± 0.1 ^c^
TVB-N * (mg·(100 g)^−1^)	12.5 ± 2.3	9.9± 0.5	20.4 ± 1.6	17.5 ± 1.3
Moisture (%)	80.2 ± 0.9 ^a^	79.0 ± 0.5 ^b^	76.6 ± 0.8 ^b^	75.7 ± 0.8 ^b^
Lipid (%)	0.8 ± 0.3 ^a^	1.2 ± 0.1 ^a^	3.8 ± 0.9 ^b^	3.2 ± 0.7 ^b^
Protein (%)	18.2 ± 0.5 ^a^	18.9 ± 0.5 ^a^	18.2 ± 0.4 ^a^	20.8 ± 0.6 ^b^
Ash (%)	1.0 ± 0	0.9 ± 0.2	1.1 ± 0.1	1.1 ± 0.1
NaCl (%)	0.2 ± 0.1	0.4 ± 0.1	0.3 ± 0.1	0.2 ± 0.1
P_2_O_5_ (g·kg^−1^)	3.4 ± 0.1 ^a^	3.5 ± 0.2 ^a,b^	4.2 ± 0.2 ^b,c^	4.3 ± 0.2 ^c^
Citric acid (g·kg^−1^)	0.19 ± 0.06	<0.02	<0.02	<0.005 ^1^
Diphosphate (g·kg^−1^)	<0.01	<0.01	<0.01	<0.01
Triphosphate (g·kg^−1^)	<0.01	<0.01	<0.01	<0.01
Na (mg·kg^−1^)	1255 ± 295 ^a^	1770 ± 79 ^a^	633 ± 38 ^b^	336 ± 42 ^c^
K (mg·kg^−1^)	2689 ± 226 ^a^	2212 ± 167 ^b^	3579 ± 173 ^c^	3858 ± 23 ^d^
Ca (mg·kg^−1^)	273 ± 24	138 ± 8	281 ± 89	227 ± 20
Mg (mg·kg^−1^)	237 ± 17	213 ± 10	295 ± 26	268 ± 19
Zn (mg·kg^−1^)	4.16 ± 0.30	4.61 ± 0.40	5.51 ± 0.38	4.39 ± 0.29
Se (mg·kg^−1^)	0.24 ± 0.08	0.29 ± 0.08	0.19 ± 0.02	0.34 ± 0.04
As (mg·kg^−1^)	0.70 ± 0.64	1.09 ± 0.27	0.14 ± 0.02	0.42 ± 0.07
*Most frequent free amino acids (FAA) and sums of FAA groups*				
Alanine (mg·(100 g)^−1^)	15.5 ± 5.6 ^a,b^	9.8 ± 1.0 ^a^	28.5 ± 3.9 ^c^	20.6 ± 6.1 ^b^
Glutamic acid mg·(100 g)^−1^)	19.0 ± 6.6 ^a^	10.8 ± 1.0 ^a^	52.3 ± 10.2 ^b^	18.2 ± 10.7 ^a^
Glycine (mg·(100 g)^−1^)	33.5 ± 36.1 ^a^	9.5 ± 1.7 ^a^	274.5 ± 26.2 ^b^	36.3 ± 13.6 ^a^
Taurine (mg·(100 g)^−1^)	212.5 ± 62.4 ^a^	169.5 ± 3.9 ^a,b^	70.2 ± 10,3 ^b^	262.4 ± 44.1 ^a^
∑ indispens. FAA ^2^ (mg·(100 g)^−1^)	28 ± 8 ^a^	20 ± 1 ^a^	33 ± 10 ^a,b^	56 ± 29 ^b^
∑ cond. indispens. FAA ^3^ (mg·(100 g)^−1^)	224 ± 62 ^a,c^	178 ± 6 ^b,c^	87 ± 13 ^b^	282 ± 44 ^c^
Total FAA ^4^ (mg·(100 g)^−1^)	328 ± 54 ^a^	235 ± 11 ^b^	519 ± 54 ^c^	430 ± 71 ^d^

^1^ *n* = 9 fish samples; ^2^ Indispensable FAA: His, Ile, Leu, Lys, Met, Phe, Trp, Thr, Val; ^3^ Conditionally indispens. FAA: Arg, Tau, Tyr; ^4^ Total FAA: Ala, Asn, Asp, Glu, Gly, Ser, Orn His, Ile, Leu, Lys, Met, Phe, Trp, Thr, Val, Arg, Tau, Tyr; * Total volatile basic nitrogen. Values with different superscripts within a line are significantly different (*p* < 0.05).

**Table 3:** Meat composition: Mean values ± standard deviation of farmed pangasius.

Species and Origin		Pangasius, Aquaculture Vietnam			
		Whole		Cutlets	Organic Fillets
Number of Samples		Skinned 10	Deep-Skinned 10	10	10
pH		6.4 ± 0.2	6.5 ± 0.2	6.6 ± 0.1	6.7 ± 0.1
TVB-N * (mg·(100 g)^−1^)		n.d.	12.4 ± 1.6	11.6 ± 0.8	11.3 ± 1.1
Moisture (%)		75.3 ±3.2 ^a^	78.9 ± 1.1 ^b^	78.6 ± 1.8 ^b^	79.7 ± 0.6 ^b^
Lipid (%)		7.8 ± 3.6 ^a^	2.7 ± 0.9 ^b^	2.4 ± 0.5 ^b^	3.5 ± 1.0 ^b^
Protein (%)		17.2 ± 1.6 ^a^	18.7 ± 1.1 ^b^	17.8 ± 1.3 ^b^	18.1 ± 0.5 ^a,b^
Ash (%)		0.8 ± 0.1	0.9 ± 0.1	0.9 ± 0.1	0.9 ± 0.1
NaCl (%)		0.1	n.d.	0.1	0.1
P_2_O_5_ (g·kg^−1^)		3.49 ± 0.71 ^a^	4.05 ± 0.69 ^a,b^	4.50 ± 1.42 ^a,b^	5.32 ± 1.41 ^b^
Citric acid (g·kg^−1^)		<0.005	<0.005	<0.005	<0.005
Diphosphate (g·kg^−1^)		<0.01 ^1^	<0.01 ^1^	<0.01 ^1^	<0.01 ^1^
Triphosphate (g·kg^−1^)		<0.01 ^1^	<0.01 ^1^	<0.01 ^1^	<0.01 ^1^
Na (mg·kg^−1^)		405 ± 128 ^a^	412 ± 144 ^a^	322 ± 92 ^a^	389 ± 52 ^a^
K (mg·kg^−1^)		3006 ± 350 ^a^	3072 ± 137 ^a^	3003 ± 43 ^a^	3132 ± 207 ^a^
Ca (mg·kg^−1^)		162 ± 28	149 ± 21	184 ± 93	127 ± 10
Mg (mg·kg^−1^)		244 ± 29	252 ± 23	249 ± 19	254 ± 14
Zn (mg·kg^−1^)		5.89 ± 1.44	4.95 ± 1.01	4.68 ± 0.36	3.67 ± 0.25
Se (mg·kg^−1^)		0.18 ± 0.02	0.18 ± 0.01	0.15 ± 0.01	0.12 ± 0.01
As (mg·kg^−1^)		<0.02	<0.02	<0.02	<0.02
*Most frequent free amino acids (FAA) and sums of FAA groups*					
Arginine (mg·(100 g)^−1^)		11.9 ± 4.4 ^a^	13.5 ± 6.1 ^a^	11.1 ± 3.5 ^a^	8.7 ± 1.3 ^a^
Glycine (mg·(100 g)^−1^)		8.3 ± 3.7 ^a^	9.4 ± 3.9 ^a,b^	14.3 ± 4.9 ^b^	8.0 ± 2.1 ^a^
Lysine (mg·(100 g)^−1^)		9.8 ± 4.9 ^a^	11.0 ± 6.0 ^a^	34.8 ± 11.2 ^b^	7.5 ± 1.5 ^a^
Taurine (mg·(100 g)^−1^)		88.0 ± 27.7 ^a^	95.5 ± 32.0 ^a^	99.9 ± 9.6 ^a^	85.9 ± 15.3 ^a^
∑ indispens. FAA ^2^ (mg·(100 g)^−1^)		32 ± 8 ^a,b^	36 ± 9 ^a^	68 ± 28 ^c^	18 ± 3 ^b^
∑ cond. indispens. FAA ^3^ (mg·(100 g)^−1^)		104 ± 25 ^a^	114 ± 29 ^a^	117 ± 9 ^a^	97 ± 16 ^a^
Total FAA ^4^ (mg·(100 g)^−1^)		171 ± 24 ^a,b^	186 ± 30 ^a^	242 ± 46 ^c^	144 ± 18 ^b^

^1^ *n* = 5 fish samples; ^2^ Indispensable FAA: His, Ile, Leu, Lys, Met, Phe, Trp, Thr, Val ^3^ Conditionally indispens. FAA: Arg, Tau, Tyr; ^4^ total FAA: Ala, Asn, Asp, Glu, Gly, Ser, Orn His, Ile, Leu, Lys, Met, Phe, Trp, Thr, Val, Arg, Tau, Tyr; * Total volatile basic nitrogen. Values with different superscripts within a line are significantly different (*p* < 0.05); n.d. = not determined.

**Table 4:** Fatty acid (FA) composition: Mean values ± standard deviation of turbot from four different aquaculture farms and wild turbot (origin North-East (NE) Atlantic), respectively (% of fatty acids measured; SFA = saturated fatty acids; MUFA = monounsaturated fatty acids; PUFA = polyunsaturated fatty acids).

Origin		Aquaculture Spain		Aquaculture Chile		NE-Atlantic Wild
Number of Fish		8	10	10	10	9
FA Common Name	FA Shorthand					
Myristic acid	14:0	5.2 ± 0.41	3.8 ± 1.00	4.4 ± 0.29	4.5 ± 0.58	3.7 ± 1.20
Pentadecanoic acid	15:0	0.4 ± 0.02	0.1 ± 0.14	0.2 ± 0.13	0.4 ± 0.13	0.5 ± 0.08
Palmitic acid	16:0	16.7 ± 0.89 ^a^	16.6 ± 1.27 ^a^	16.9 ± 0.94 ^a^	17.5 ± 1.39 ^a^	16.9 ± 1.65 ^a^
Heptadecanoic acid	17:0	0.1 ± 0.13	0.0 ± 0.00	n.d.	0.1 ± 0.17	0.2 ± 0.19
Stearic acid	18:0	3.5 ± 0.54	4.3 ± 0.89	3.5 ± 0.38	4.0 ± 0.55	3.8 ± 0.88
	**∑SFA**	**25.8**	**24.9**	**25.0**	**26.4**	**25.2**
Palmitoleic acid	16:1n-7	6.3 ± 0.63	5.2 ± 1.36	5.9 ± 0.48	5.4 ± 0.85	4.4 ± 0.83
Oleic acid	18:1n-9*c*	12.3 ± 0.41 ^a^	11.6 ± 0.93 ^a^	11.8 ± 0.45 ^a^	11.5 ± 0.78 ^a^	9.8 ± 0.85 ^b^
Vaccenic acid	18-1n-7	3.1 ± 0.09	3.0 ± 0.10	2.9 ± 0.06	2.9 ± 0.14	2.7 ± 0.52
Gondoic acid	20:1n-9	1.4 ± 0.11 ^a^	1.1 ± 0.30 ^a^	1.5 ± 0.14 ^a^	1.4 ± 0.13 ^a^	3.8 ± 1.94 ^b^
Erucic acid	22:1n-9	0.3 ± 0.04	0.1 ± 0.13	0.2 ± 0.10	0.2 ± 0.16	0.6 ± 0.29
	**∑MUFA**	**23.4**	**21.0**	**22.4**	**21.4**	**21.3**
Linoleic acid	18:2n-6*c*	6.6 ± 0.25 ^a^	3.3 ± 0.23 ^b^	3.8 ± 0.12 ^a^	6.5 ± 0.46 ^a^	1.5 ± 0.28 ^b^
γ-Linolenic acid	18:3n-6	0.0 ± 0.00	0.0 ± 0.00	n.d.	n.d.	0.0 ± 0.00
α-Linolenic acid	18:3n-3	0.9 ± 0.09	0.6 ± 0.11	0.7 ± 0.05	0.8 ± 0.13	0.7 ± 0.21
Stearidonic acid	18:4n-3	1.4 ± 0.18	1.2 ± 0.39	1.4 ± 0.13	1.1 ± 0.23	1.2 ± 0.41
Eicosadienic acid	20:2n-6	0.6 ± 0.04	0.2 ± 0.13	0.2 ± 0.18	0.6 ± 0.07	0.5 ± 0.04
Arachidonic acid	20:4n-6	1.6 ± 0.16	1.7 ± 0.36	1.7 ± 0.28	2.0 ± 0.39	2.4 ± 0.62
Eicosapentaenoic acid (EPA)	20:5n-3	10.8 ± 0.45 ^a^	12.6 ± 0.41 ^b^	12.4 ± 0.54 ^b^	10.1 ± 0.67 ^a^	6.3 ± 0.53 ^c^
Docosatetraneoic acid	22:4n-6	n.d.	0.3 ± 0.14	0.2 ± 0.13	0.2 ± 0.14	0.4 ± 0.25
Docosapentaenoic acid (DPA)	22:5n-3	4.2 ± 0.16	5.1 ± 0.40	4.5 ± 0.26	4.1 ± 0.29	3.5 ± 0.76
Docosahexaenoic acid (DHA)	22:6n-3	13.6 ± 1.86 ^a^	17.6 ± 3.37 ^b^	16.1 ± 1.44 ^a^	15.9 ± 1.89 ^a^	22.6 ± 2.68 ^c^
	**∑PUFA**	**39.7**	**42.6**	**40.9**	**41.3**	**39.1**
	unidentified	11.1	11.6	11.7	11.0	14.4
	∑n-3	30.9	37.1	35.0	32.0	34.3
	∑n-6	8.8	5.5	6.0	9.3	4.8
	Ratio n-3/n-6	3.5	6.8	5.9	3.5	7.1
	EPA ± DHA	24.4	30.2	28.5	26.0	28.8

n.d. = not detected; Values with different superscripts within a line are significantly different (*p* < 0.05).

**Table 5:** Fatty acid (FA) composition: Mean values ± standard deviation of farmed and wild barramundi of different origins (% of fatty acids measured; SFA = saturated fatty acids; MUFA = monounsaturated fatty acids; PUFA = polyunsaturated fatty acids).

	Species and Origin	Barramundi Vietnam		Barramundi Australia	
Number of Samples		Aquaculture 10	Wild 4	Aquaculture 10	Wild 10
FA Common Name	FA Shorthand				
Myristic acid	14:0	3.5 ± 1.06	3. 5 ± 0. 62	2.8 ± 0.06	4.1 ± 0.38
Pentadecanoic acid	15:0	1.1 ± 0.85 ^a^	0.7 ± 0.11 ^a^	0.3 ± 0.01 ^b^	4.2 ± 0.29 ^c^
Palmitic acid	16:0	22.8 ± 2.43 ^a^	26.6 ± 1.13 ^b^	19.3 ± 0.47 ^a^	26.9 ± 0.61 ^b^
Heptadecanoic acid	17:0	1.2 ± 0.69	0.8 ± 0.08	0.3 ± 0.10	1.6 ± 0.14
Stearic acid	18:0	8.3 ± 2.61	9.5 ± 0.40	5.4 ± 0.26	6.7 ± 0.57
	∑SFA	36.9	41.0	28.0	43.5
Palmitoleic acid	16:1n-7	5.2 ± 1.95 ^a^	4.6 ± 0.41 ^b^	6.6 ± 0.14 ^c^	9.6 ± 0.77 ^d^
Oleic acid	18:1n-9*c*	15.6 ± 5.44 ^a^	17.3 ± 2.52 ^a^	33.7 ± 0.53 ^b^	12.8 ± 0.73 ^a^
Vaccenic acid	18-1n-7	3.0 ± 0.58	2.5 ± 0.09	2.6 ± 0.04	2.5 ± 0.06
Gondoic acid	20:1n-9	0.8 ± 0.31 ^a^	0.4 ± 0.06 ^b^	0.6 ± 0.03 ^a,b^	0.7 ± 0.03 ^a^
	∑MUFA	24.6	24.8	43.6	25.6
Linoleic acid	18:2n-6*c*	5.0 ± 4.27 ^a^	1.7 ± 1.04 ^b^	10.9 ± 0.16 ^a^	1.6 ± 0.14 ^b^
γ-Linolenic acid	18:3n-6	0.2 ± 0.16	0.0 ± 0.00	0.4 ± 0.04	0.6 ± 0.04
α-Linolenic acid	18:3n-3	1.1 ± 0.34	0.5 ± 0.08	1.5 ± 0.06	1.9 ± 0.07
Stearidonic acid	18:4n-3	0.6 ± 0.33	0.3 ± 0.17	0.6 ± 0.03	0.8 ± 0.07
Eicosadienic acid	20:2n-6	0.3 ± 0.16	0.1 ± 0.11	0.1 ± 0.09	0.0 ± 0.00
Arachidonic acid	20:4n-6	3.3 ± 3.55	3.3 ± 0.57	0.7 ± 0.14	3.4 ± 0.24
Eicosapentaenoic acid (EPA)	20:5n-3	3.0 ± 1.06 ^a^	2.5 ± 0.20 ^b^	3.0 ± 0.11 ^a^	3.3 ± 0.17 ^a^
Docosatetraneoic acid	22:4n-6	0.7 ± 0.54	0.8 ± 0.30	0.0 ± 0.00	0.9 ± 0.17
Docosapentaenoic acid (DPA)	22:5n-3	2.6 ± 0.39	2.4 ± 0.24	1.0 ± 0.07	1.8 ± 0.15
Docosahexaenoic acid (DHA)	22:6n-3	9.2 ± 4.75 ^a^	13.3 ± 1.29 ^a^	3.1 ± 0.33 ^b^	4.1 ± 0.31 ^a,b^
	∑PUFA	26.0	25.0	21.2	18.5
	unidentified	12.6	9.2	7.2	12.4
	∑n-3	16.4	19.0	9.2	11.9
	∑n-6	9.5	5.9	12.0	6.6
	Ratio n-3/n-6	1.7	3.2	0.8	1.8
	EPA + DHA	12.2	15.8	6.2	7.4

Values with different superscripts within a line are significantly different (*p* < 0.05).

**Table 6:** Fatty acid (FA) composition: Mean values ± standard deviation of farmed pangasius (% of fatty acids measured; SFA = saturated fatty acids; MUFA = monounsaturated fatty acids; PUFA = polyunsaturated fatty acids).

Species and Origin		Pangasius, Aquaculture Vietnam				
			Whole		Cutlets	Organic Fillets
Number of Samples			Skinned 10	Deep-Skinned 10	10	10
FA Common Name		FA Shorthand				
Myristic acid		14:0	3.7 ± 0.89	3.4 ± 0.96	4.0 ± 0.31	3.5 ± 0.29
Pentadecanoic acid		15:0	0.4 ± 0.23	0.3 ± 0.26	0.1 ± 0.09	0.02 ± 0.06
Palmitic acid		16:0	28.9 ± 1.50 ^a,b^	27.9 ± 2.16 ^a,b^	29.8 ± 0.66 ^b^	27.7 ± 0.65 ^a^
Heptadecanoic acid		17:0	0.5 ± 0.37	0.5 ± 0.42	0.1 ± 0.10	n.d.
Stearic acid		18:0	9.9 ± 1.17	10.1 ± 1.17	8.6 ± 0.51	9.1 ± 0.37
		**∑SFA**	**43.4**	**42.2**	**42.5**	**40.3**
Palmitoleic acid		16:1n-7	1.6 ± 0.32	1.5 ± 0.25	1.0 ± 0.09	0.8 ± 0.05
Oleic acid		18:1n-9*c*	29.0 ± 6.43 ^a^	27.8 ± 6.7 ^a^	35.7 ± 0.66 ^b^	31.1 ± 0.99 ^c^
Vaccenic acid		18-1n-7	1.5 ± 0.50	1.6 ± 0.59	0.8 ± 0.06	0.8 ± 0.03
Gondoic acid		20:1n-9	1.1 ± 0.22 ^a^	1.1 ± 0.2 ^a^	1.1 ± 0.1 ^a^	1.1 ± 0.05 ^a^
		**∑MUFA**	**33.3**	**32.0**	**38.7**	**33.8**
Linoleic acid		18:2n-6*c*	4.4 ± 2.47 ^a^	4.3 ± 2.48 ^a^	8.5 ± 0.58 ^b^	13.5 ± 0.48 ^b^
γ-Linolenic acid		18:3n-6	n.d.	n.d.	0.2 ± 0.15	0.1 ± 0.11
α-Linolenic acid		18:3n-3	0.5 ± 0.14	0.5 ± 0.17	0.5 ± 0.05	1.1 ± 0.06
Stearidonic acid		18:4n-3	0.04 ± 0.00	0.0 ± 0.00	0.0 ± 0.00	0.0 ± 0.00
Eicosadienic acid		20:2n-6	0.5 ± 0.09	0.5 ± 0.11	0.5 ± 0.06	0.8 ± 0.07
Arachidonic acid		20:4n-6	1.3 ± 0.79	1.9 ± 1.02	1.0 ± 0.23	1.3 ± 0.19
Eicosapentaenoic acid (EPA)		20:5n-3	1.2 ± 0.79 ^a^	1.2 ± 0.77 ^a^	0.1 ± 0.09 ^b^	0.2 ± 0.08 ^b^
Docosatetraneoic acid		22:4n-6	0.2 ± 0.23	0.2 ± 0.25	0.1 ± 0.13	0.2 ± 0.17
Docosapentaenoic acid (DPA)		22:5n-3	0.8 ± 0.42	0.8 ± 0.41	0.1 ± 0.12	0.4 ± 0.05
Docosahexaenoic acid (DHA)		22:6n-3	6.4 ± 5.23 ^a^	7.8 ± 5.89 ^a^	0.6 ± 0.13 ^b^	1.4 ± 0.19 ^c^
		**∑PUFA**	**15.3**	**17.3**	**11.7**	**18.8**
		unidentified	8.0	8.5	7.2	7.1
		∑n-3	8.9	10.3	1.3	3.0
		∑n-6	6.5	7.0	10.3	15.7
		Ratio n-3/n-6	1.4	1.5	0.1	0.2
		EPA ± DHA	7.6	8.9	0.7	1.6

n.d. = not detected.
